# A Comparison of the Sticky Bone Obliteration Technique and Obliteration Using S53P4 Bioactive Glass After Canal Wall Down Ear Surgery: A Preliminary Study

**DOI:** 10.3390/jcm14051681

**Published:** 2025-03-01

**Authors:** Aleksander Zwierz, Marta Staszak, Matthias Scheich, Krzysztof Domagalski, Stephan Hackenberg, Paweł Burduk

**Affiliations:** 1 Department of Otolaryngology, Phoniatrics and Audiology, Faculty of Medicine, Ludwik Rydygier Collegium Medicum, Nicolaus Copernicus University, 85-067 Bydgoszcz, Poland; mart.staszak@gmail.com (M.S.); pburduk@wp.pl (P.B.); 2 Department of Otorhinolaryngology, Plastic, Aesthetic and Reconstructive Head and Neck Surgery, University Hospital Würzburg, 97080 Würzburg, Germany; scheich_m@ukw.de (M.S.); hackenberg_s@ukw.de (S.H.); 3 Department of Immunology, Faculty of Biological and Veterinary Sciences, Nicolaus Copernicus University, 87-100 Torun, Poland; krydom@umk.pl

**Keywords:** canal wall down surgery, CWD, mastoid obliteration, cholesteatoma, sticky bone, bioactive glass

## Abstract

**Background:** The aim of this study was to analyse the results of the mastoid obliteration technique with sticky bone (SB) and compare them with those obtained using bioactive glass S53P4 (BAG). **Methods:** This prospective preliminary study comprised 28 adults who underwent canal wall down (CWD) surgery using two mastoid obliterative techniques: SB (*n* = 21) or BAG (*n* = 7). The SB group was treated with the patients’ own bone dust and injectable platelet rich fibrin (IPRF) (*n* = 13%) or bone dust, IPRF, and additionally allogenic lyophilised demineralised bone (*n* = 9%). **Results:** Nine months after the surgery, in the SB group, retroauricular depression was observed in three (14%) patients, temporary retroauricular fistula in one (5%), and a conical and smooth external auditory canal (EAC) was achieved in 15 (71%). Mean EAC capacity was 0.6 mL higher than in the contralateral ear. In the SB group, the tympanic membrane (TM) of nineteen (91%) patients was fully healed, one (5%) had TM perforation, and one (5%) developed a retraction pocket. In the BAG group, retroauricular depression was observed in four (57%) patients, temporary retroauricular fistula was present in one (14%), and a conical and smooth EAC was achieved in five (71%). Mean EAC capacity was 0.3 mL higher than on the opposite side. In the BAG group, we stated six (86%) patients with fully healed TM and one (14%) with a retraction pocket. One cholesteatoma was found in the BAG group and two in SB, (14% vs. 10%). After 9 months, all patients in both groups achieved a dry and self-cleaning cavity. **Conclusions:** Mastoid obliteration in CWD surgery using SB or BAG allows for reconstruction of the conical shape of the EAC with a volume similar to that of a healthy ear. Both techniques seem to have a minimal risk of complications and result in a dry, self-cleaning cavity. Further studies concerning a larger series of cases are necessary to confirm the findings of this preliminary analysis.

## 1. Introduction

In recent decades, two main operating techniques have predominated in the treatment of cholesteatoma. The first involves maintaining the natural anatomical conditions of the ear including the posterior wall of the external auditory canal (EAC) and performing a mastoidectomy, a posterior tympanotomy to remove the cholesteatoma from the double approach (through EAC and trans mastoid). The second involves performing a mastoidectomy, removing the posterior wall of the EAC, and modifying the anatomy for better access to the middle ear pathology. Each of these operating techniques has advantages and disadvantages. However, it is not always possible to preserve the posterior wall. In some cases, advancement of the pathological process in the ear has already caused such damage to the posterior wall that the surgeon has no choice but to remove it. After that, there are again two surgical options: leaving a large postoperative cavity connecting the EAC with the mastoid cavity (“radical cavity”) or attempting to reconstruct the posterior wall of the EAC. Leaving an open cavity entails the need for regular cleaning and micro suction. It also increases the risk of crust accumulation, water intolerance, water- or air-induced vertigo, and discharge. However, many ENT surgeons have employed this surgical technique for decades when performing cholesteatoma removing surgery as it allows for good control of the middle ear pathology and ease in anatomical conditions.

The concept of obliteration using a muscle flap was first introduced by Moscher in 1911 to restore physiological/anatomical conditions of the ear [1]. In 1987, Mercke used bone dust to fill the mastoid cavity in cholesteatoma surgery [2]. Since then, a variety of reconstructive materials have been used to restore middle ear and mastoid anatomy, all of which have advantages and disadvantages. Generally, two groups of reconstructive materials are used: local flaps (meatally based musculoperiosteal flap [Palva flap], middle temporal artery flap, Hong Kong flap, temporoparietal fascial flap, pedicled superficial temporalis fascial flap, postauricular-periosteal-pericranial flap, temporalis muscle flap, inferiorly based fascio-periosteal flap, postauricular myocutaneous flap) or materials that enable restoration of the rigid posterior wall (bone pate, bioactive glass [BAG]-S53P4 or bioactive glass-45S5, titanium, silicone) [3,4,5,6,7,8,9,10,11,12,13,14,15,16,17]. Due to the good blood supply of muscle flaps, good healing can be achieved by using them as a reconstructive material. However, muscle flaps may shrink during tissue healing. Thus, anatomical conditions of the EAC may not be restored fully, leading to cavity depressions. Although bone materials and their analogues can fill the postoperative cavity, these materials can become infected, especially when they are used in a chronically inflamed ear. Autogenous materials available during the procedure may not be sufficient to replace tissue loss. Therefore, biocompatible materials, such as BAG, hydroxyapatite, titanium, and silicon block, or allogeneic materials, such as demineralized or cancellous bone, have been introduced for complete filling of the cavity [8,9,10,11,12,13,14,15,18]. In 2009, Lee et al. presented an interesting concept of canal wall reconstruction and mastoid obliteration using autogenous bone pate mixed with 3–5 mm sized allogenous cancellous bone chips [7] and achieved a cylindrical-shape EAC in 90% of patients. Unfortunately, in the postoperative period, retroauricular skin inflammation occurred in 13.6% patients and fistula formation occurred in 9.1% of patients. To optimise this method of mastoid obliterative treatment, we decided to slightly modify the obliteration material. We used autogenous bone dust mixed with allogenic lyophilised demineralised bone and injectable plate rich fibrin (IPRF+) to obtain sticky bone and protected the graft by covering it with an advanced platelet-rich fibrin (APRF) membrane. IPRF is commonly used in different types of maxillofacial and dental surgeries to replace bone defects [19,20]. It has a number of advantageous characteristics such as fibroblast growth factors that induce tissue regeneration as well as anti-inflammatory and antimicrobial activity [19,20,21,22,23,24,25].

The aim of this study was to analyse results obtained using sticky bone as an obliteration method, especially in terms of its effectiveness, minimisation of complications, and accuracy of EAC reconstruction, and to compare the results with those obtained with obliteration using BAG-S53P4 (BonAlive Biomaterials Ltd., Turku, Finland).

## 2. Materials and Methods

This prospective study comprised 28 patients,12 females, and 16 males aged 17–77 years (mean age: 45.6 years) treated surgically between 2022 and 2023 for chronic otitis media with cholesteatoma using one of two mastoid obliterative techniques: sticky bone or BAG-S53P4 as a reference technique. The chosen obliterative technique was selected randomly. During each procedure, we had full availability of all obliterative materials and needed instruments, but in different quantities. Therefore, we prepared lottery tickets, where we had written BAG (bioactive glass) on eight pieces, BD (bone dust) on twelve pieces, and BD and DB (bone dust and demineralised bone) on twelve pieces. During the surgery, when the decision was made about canal wall down surgery, the instrumentalist drew lots indicating the material used for obliteration. All procedures were performed by the same surgeon (AZ). There were seven patients in the BAG group and 21 in the sticky bone group with two subgroups: twelve (57%) patients were treated with bone dust collected during the surgery and IPRF, and nine (43%) patients were treated with bone dust collected during the surgery, IPRF, and allogenic lyophilised demineralised bone. One additional patient was excluded from the studies due to prior surgery of the contralateral ear, which made it impossible to perform a comparative EAC volumetric examination.

### 2.1. Surgical Technique

**Figure 1:**
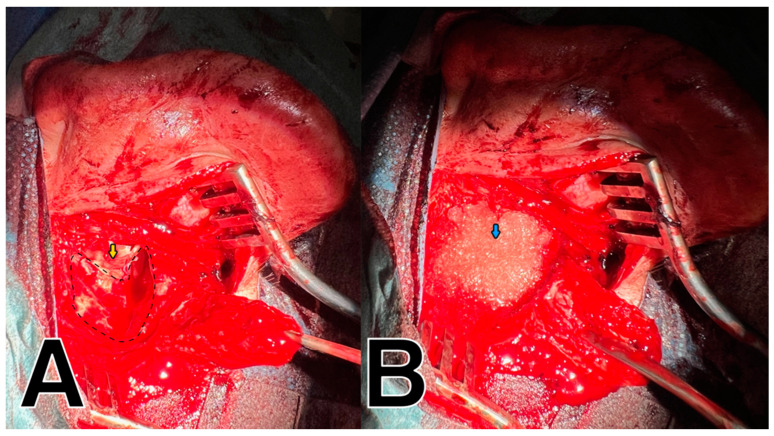
Mastoid obliterative technique with the use of bioactive glass—left ear. (**A**). Mastoid cavity before obliteration—dashed line. Cartilage and fascia constituting scaffolding for the EAC—yellow arrow (**B**). Mastoid obliterated with bioactive glass—blue arrow.

**Figure 2:**
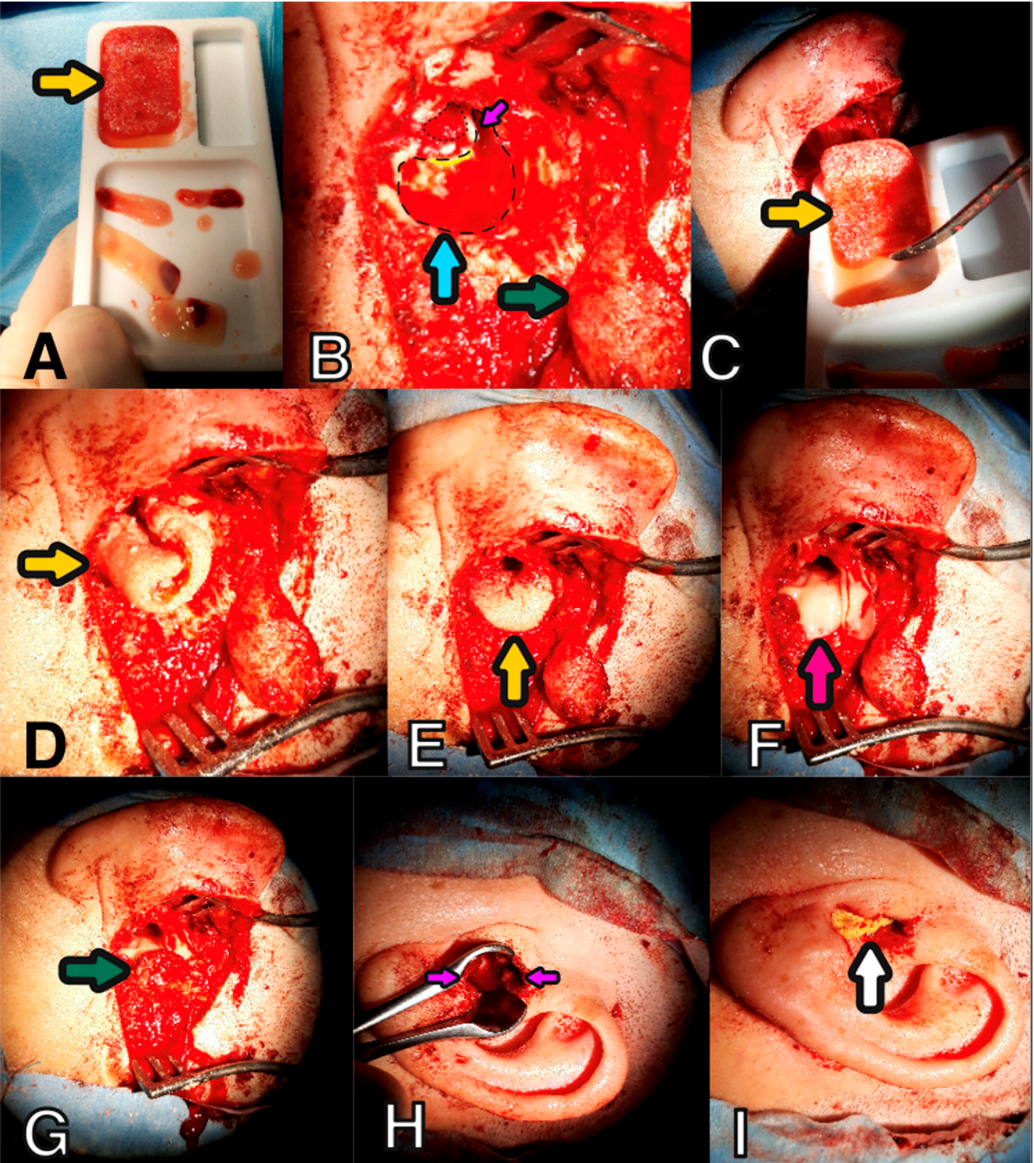
Mastoid obliterative technique with use of sticky bone—left ear. (**A**) Preparation of sticky bone—yellow arrow. (**B**) Mastoid cavity before obliteration—dashed line; TM covered with spongostan—dotted line; facial nerve—yellow line; posterior margin of the mastoid cavity—blue arrow; up-pedunculated muscle flap—green arrow. Cartilage used to attic stabilisation and obliteration—violet arrow. (**C**) Sticky bone moved to the mastoid cavity—yellow arrow. (**D**) Rolled sticky bone matched to the mastoid cavity—yellow arrow. (**E**) Final placement of sticky bone—yellow arrow. (**F**) Sticky bone covered with APRF—pink arrow (**G**) Up-pedunculated muscle flap moved onto the top of the obliterated cavity—green arrow. (**H**) Place of the of the EAC cartilage incision—violet arrow. (**I**) EAC packed with cotton pledge with antibiotic ointment—white arrow.

In all patients, a retroauricular approach was adopted during the surgical procedure performed to remove the cholesteatoma from the tympanic cavity and/or mastoid cavity. After cutting the skin and subcutaneous tissue, an up-pedunculated muscle flap was harvested. In the case of a first-time surgery, mastoidectomy was performed, the facial nerve was identified, and the posterior wall of the EAC was removed. The tympanic membrane was lifted, and the cholesteatoma was cleaned from the tympanic cavity. In cases of reoperation where the posterior wall had been previously preserved, the posterior wall of the EAC was removed, and the cholesteatoma was then removed from the mastoid and tympanic cavity. In cases of reoperation after the canal wall down (CWD) procedure, the healthy skin covering the cavity was lifted, and cholesteatoma masses, granulation tissues, and epidermis were removed from the postoperative mastoid and tympanic cavity. In all cases of destruction of the eardrum or the ossicular chain, a tympanic membrane reconstruction was performed using the perichondral or temporal muscle fascia, and the ossicular chain was reconstructed with a titanium total ossicular replacement prothesis (TORP) or partial ossicular replacement prothesis (PORP) (Grace Medical, Memphis, TN, USA). The tympanoplasty was performed in accordance with the Wullstein classification [26]. The upper and posterior walls of the small tympanic cavity were closed with cartilage taken from the auricle. The mastoid cavity and epitympanum were randomly filled with sticky bone or BAG. Prior to surgery, a variant of the obliterative technique was drawn and the patient was informed and approved the treatment. In the BAG group, the temporal facia and cartilage were used to cover the used obliterative material from the EAC side (Figure 1). In the obliteration with the sticky bone group, an APRF membrane was also used (Figure 2). The remnants of the skin of the EAC covered the fascia and APRF membrane. In addition, to slightly widen the entrance to the EAC, an incision was made in the skin and cartilage of the EAC at the 6 and 12 o’clock positions. The eardrum graft was covered with a round fragment of thin sponge (Spongostan^®^; Ethicon, Johnson & Johnson MedTech, Raritan, NJ, USA), and the EAC was covered with thin silastic strips and filled with a cotton pledge soaked in antibiotic ointment. The tissue behind the ear was sutured in layers, and a pedunculated muscle flap was placed at the top of the obliterative material in place of the performed obliteration.

### 2.2. Sticky Bone

The proposed material (sticky bone) is a mixture of bone dust and a solution of IPRF+. Two types of bone were used: autologous bone dust collected during surgery using a bone dust collector (Omnia srl, Fidenza, Italy) and allogenic lyophilised demineralised cortical bone. In 12 (57%) patients of the sticky bone group, only the patient’s own material was used for obliteration, with sticky bone produced from bone dust collected during the surgery and IPRF+. In the other nine (43%) patients of the sticky bone group, this mixture was supplemented with lyophilised demineralised cortical bone to achieve the optimal amount of obliterative material.

The procedure was as follows. Shortly before the end of the surgery, a small amount of blood (40 mL) was drawn from the patient and centrifuged in four sterile 13 mL IPRF+ tubes at 700 rpm for 5 min (Duo^®^ Quattro PRF, Avtec Surgical, LLC, Mt Pleasant, SC, USA). The superficial portion of the fluid from the tubes was then collected using a syringe, which yielded about 3–4 mL of IPRF+. After 20 min, the bony dust combined with liquid IPRF+ formed a sticky bone with a jelly-like consistency, which was used for obliteration. Plasma was then separated from the four tubes, each containing 10 mL of blood and centrifuged at 1300 rpm for 8 min. The resulting APRF had a high concentration of platelets (for clotting) and white blood cells (for immune defence). After collection, the APRF was flattened and formed into membranes used to create autologous wound dressings.

### 2.3. Postoperative Management

All patients were discharged from the clinic the day after surgery. Systemic antibiotic therapy was continued for 7 days for all patients. In cases of normal healing, the retroarticular sutures were removed during follow-up 7 days later, and the cotton pledge with antibiotic ointment and silastic strips were removed during a follow-up visit 2 weeks later. For the next 3 weeks, the patients were instructed to instil three drops of antibiotic drops three times daily into the EAC. Five weeks after the procedure, a follow-up otomicroscopy with cleaning of the postoperative cavity was performed. Follow-up examinations were then performed at 3, 6, and 9 months. In accordance with study protocol, a follow-up computed tomography (CT) scan of the temporal bone was performed 6–9 months after the procedure. The postoperative cavity was assessed during the follow-up visits according to the middle ear post-operative infection grading classification proposed by Merchant et al. [27].

### 2.4. Evaluation of Treatment

During the follow-up examinations, retroauricular wound healing was assessed and the fistula formation inspected. The skin regeneration of the EAC and the self-cleaning ability of the postoperative cavity were also assessed by observation of a cerumen and epidermis accumulation. In addition, the EAC capacity of the operated ear compared with the non-operated ear was assessed by measuring in millilitres the volume of water needed to fill the EAC to the level of the concha while the patient lay in a supine position on the couch with their head rotated.

### 2.5. Study Group

The study group comprised twenty-eight patients treated for cholesteatoma: seven (25%) treated with BAG-S53P4 obliteration and twenty-one (75%) with sticky bone. In the sticky bone treatment group, twelve (57%) patients were treated with bone dust collected during the surgery and IPRF, and nine (43%) patients were treated with bone dust collected during the surgery, IPRF, and allogenic lyophilised demineralised bone. Among the surgeries performed, ten were first-time surgeries, fourteen were reoperations after posterior wall preservation surgeries with residual or recurrent cholesteatoma, and four were revisions after CWD surgery. In 17 patients, the left ear was operated on, and the right ear was operated on in 11 patients. Type 1 tympanoplasty was performed in one patient, type 2 in nine patients, type 3 in fifteen patients, and type 4 in three patients, according to Wullstein’s classification (Table 1) [26]. Table 1 also provides information on the socio-demographics of the patients and group classifications, depending on the material used for obliteration, in addition to the average volume of material used for obliteration.

### 2.6. Statistical Analysis

For the descriptive statistics, quantitative variables were presented as the means ± standard deviation (SD), while categorical variables were summarised using frequency counts and percentages. Statistical significance was determined using the chi-square method or Fisher’s exact test for categorical variables and the Student’s *t*-test or a one-way ANOVA for quantitative variables to assess differences between the groups. The Shapiro–Wilk test was used to assess normality. In all of these tests, two-tailed *p*-values were used, and differences at the level of *p* < 0.05 were considered significant. All statistical analyses were performed using SPSS (Statistical Package for the Social Sciences, version 28, Armonk, NY, USA) software.

### 2.7. Ethics

This study was approved by the ethics committee of Nicolaus Copernicus University (approval no: KB334/2022, approval date: 21 June 2022).

## 3. Results

Among the 28 patients (21 in sticky bone group and 7 in the BAG group) treated surgically in our ENT department, a smooth retroauricular area was achieved in 21 (75%) cases, and a retroauricular depression was observed in 7 (25%) cases. However, the depression was more common in the patients in whom obliteration was performed using BAG-S53P4 (57%) than in those in whom the obliteration material was sticky bone (14%) (in subgroup: IPRF mixed with bone dust or bone dust mixed with demineralised bone, 8% and 22%, respectively) (Table 2). This difference was statistically significant (*p* = 0.043). In two cases, one in each study group, a cutaneous fistula was observed in the postoperative wound in the retroauricular area, which healed successfully in both cases (Table 2). In 20 (71%) patients, the EAC was conical and smooth. A hole was found in the EAC in seven (33%) out of all patients, and six cases (29%) in the sticky bone group versus one (14.3%) case in the BAG-S53P4 group (Table 2). In 25 (89%) patients in the two treatment groups, the eardrum was healed (91% vs. 86% in the sticky bone and BAG groups, respectively). After the procedure, there was one case (5%) of perforation of the tympanic membrane (TM) in the sticky bone group. There were two cases (7%) of attic retraction pockets, one (14%) patient in the BAG-S53P4 group, and one (5%) patient in the sticky bone group (bone dust only used for obliteration) (Table 2). In both cases, there was an accumulation of epidermal debris and cholesteatoma recurrence and these patients were operated on again. A third surgery was also performed in a patient in whom perforations of the eardrum were found in follow-up examinations. In this case, the residual cholesteatoma was found and removed. In total, in the follow-up examinations, cholesteatoma residue or cholesteatoma recurrence was found in 3 out of the 28 patients (11%).

**Figure 3:**
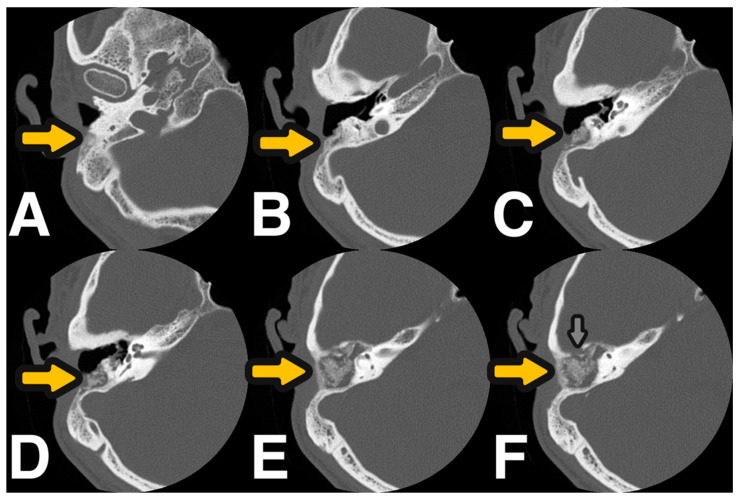
(**A**–**F**) Control HRCT-transverse plane scans of the right ear performed six months after mastoid obliteration surgery with use of sticky bone—yellow arrow. (**F**). Middle fossa bone destruction covered with sticky bone—grey arrow.

**Figure 4:**
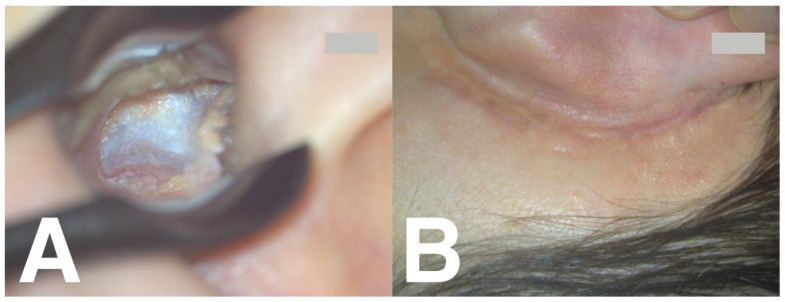
(**A**) Control otomicroscopy nine months after left ear sticky bone obliterative mastoid surgery. (**B**) Left ear retroauricular area view.

Analysing the average volume of the reconstructed EAC in the whole study sample showed that it was slightly larger in the operated than in the non-operated ears. The smallest difference in the average volume of the reconstructed EAC was found in the BAG-S53P4 group (0.3 mL vs. 0.6 mL of sticky bone composed of bone dust and IPRF or bone dust plus demineralised bone and IPRF, respectively) (Table 2), but this difference was not statistically significant. Figure 3 and Figure 4 present images from the follow-up CT and otoscopy 9 months after cavity obliteration using sticky bone composed of bone dust and lyophilised demineralised bone.

## 4. Discussion

Restoring the anatomical shape of the EAC by performing mastoid obliteration during CWD surgery or revision after previously performed CWD surgery not only improves the aesthetic appearance of the EAC, but facilitates its self-cleaning [28]. In addition to improving hearing, it makes it possible to participate actively in sports and leads to an overall improvement in quality of life [28]. According to Zwemstra et al., the best quality of sound perception is favourable in a normal-shaped ear canal [29]. A retrospective analysis by van der Toom et al. found no significant difference in the air bone gap between patients treated with CWD or CWD with mastoid obliteration [30]. Compared with the CWD procedure, CWD with obliteration enabled the isolation of the posterior labyrinth, preventing dizziness and reducing the frequency of visits to the ENT clinic. It also allowed for easier fitting of a hearing aid [30].

### 4.1. Optimal EAC Shape and Filling

The smooth conical shape of the EAC and complete filling of the obliterated cavity are extremely important for effective EAC cleaning. Obtaining a reconstructed ear canal with the correct shape is possible by using materials with high plasticity such as BAG, bone dust, or the proposed sticky bone. To fill the entire postoperative cavity, an appropriate volume of obliterative material is necessary. When using the patient’s own material, there is usually a problem with their adequacy and high availability, which poses a problem, unlike when using non-biological materials. The use of sticky bone means that an appropriate volume of autogenous obliterative material can be obtained [16]. Clinical and histopathological observations have shown that muscle flaps undergo some degree of atrophy, as confirmed in an analysis of Kim et al., which revealed retroauricular depression in the Palva flap obliterative technique after CWD surgery [4,31,32].

The high plasticity of the sticky bone used in this study filled the epitympanum, which is usually difficult when muscle flaps are used. Linthicum confirmed that grafted autologous bone pate was transformed into new bone [32]. The BAG used in our study, S53P4, retained its non-shrinking properties, unlike BAG-45S5, which was rapidly absorbed. The findings of the present study are in accordance with those of Król et al. and Sousur et al., who obtained a smooth EAC without hidden pouches in CWD surgery using BAG-S53P4 as the obliteration material [13,15].

Restoration of the original physiological volume and shape of the EAC is desirable during surgery using appropriate obliteration materials and surgical techniques in CWD surgery. There are plenty of obliteration materials available such as BAG or sticky bone composed of autogenous lyophilised demineralised cortical bone. In the present study, a good functional effect and no leakage from the postoperative cavity were found in follow-up examinations when sticky bone composed of autologous bone was used, even when the ear canal volume of the operated ear differed from the original volume.

Among the 28 patients, a conical and smooth EAC was achieved in 71% of cases. In the patients treated with sticky bone from bone dust and demineralised bone, this was achieved in 78% of cases.

It is difficult to compare the mean volume of material used for obliteration because the amount of material needed depends on anatomical conditions including aeration of the mastoid process. However, on average, about 2.8 mL was necessary to fill the cavity. The use of the above-mentioned amount of obliterative material resulted in only a 0.6 mL bigger EAC than on the non-operated ear. The EAC volume of the operated ear was similar to that of the non-operated ear in the BAG-S53P4 treatment group. Although there were fewer EACs with holes or depressions when using BAG-S53P4, there was no statistically significant difference between the BAG and the sticky bone treatment groups. However, it should be pointed out that the BAG-S53P4 group was statistically more likely to have retroauricular depression than in the sticky bone group (Table 2), but this should be confirmed on a larger group of patients. Table 1 shows a statistically significant difference in the volume of the obliterative material used in each group, which seems logical, taking into account the fact that one of the methods used only the patients’ own material and the others were supported by artificial or allogenic materials.

### 4.2. Anti-Inflammatory Effect and Surgical Healing

Obliterative materials used in an environment where there is chronic inflammation must have anti-inflammatory properties. In the case of muscle flaps, a good blood supply allows white blood cells to be delivered to the obliterated cavity to nourish the flap, thereby combatting necrosis [31]. The anti-inflammatory properties of BAG have been previously described [33]. In one study, better healing was achieved when BAG was mixed with the patient’s venous blood [13]. Bone chips used alone conferred no anti-inflammatory properties. Some authors have pointed to a high rate of superinfection and reabsorption when only bone chips were used for obliteration and recommended that bone chips should be stored in an antibiotic (rifamycin) solution prior to use [34]. Sticky bone, which is a mixture of bone and IPRF, has anti-inflammatory properties. IPRF contains leukocytes and can be a biocarrier for antibiotics. It can stimulate the growth of target cells and wound healing through the slow release of platelet-derived growth factor (PDGF) and help maintain connective tissue homeostasis [35]. PDGF initiates chemotaxis (i.e., directed cell movement) and cell shape through the reorganisation of the actin filament system. PDGF also stimulates the differentiation of specific cell types and promotes cell survival, accelerating wound healing [35].

### 4.3. Postoperative Outcomes

In the present study, at the 9-month follow-up, no discharge from the ear was found, clearly indicating a favourable impact of the materials used to reduce the volume of the postoperative cavity. Although the BAG-S53P4 patient group size in our study was small, our findings are consistent with those of De Veij Mestdagh et al., who described leakage in only 4% of patients obliterated with BAG-S53P4 in a larger sample of 67 patients [36]. Shokry et al. used BAG-45S5 for obliteration and reported infections in the postoperative cavity in 10% of patients [37]. Elbary et al. used PRF mixed with autologous bone in the reconstruction of the posterior meatal wall after CWD mastoidectomy [38]. Postoperatively, they reported temporary otorrhea in only two ears, which was controlled with antibiotic therapy, and no leakage during a follow-up of 12–24-months [38]. In the present study, among the 28 patients, inflammation of the wound behind the auricle was observed in two patients in the early postoperative period: in one patient treated with BAG-S53P4 and in one patient treated with sticky bone composed of IPRF and autologous bone dust. In both cases, wound healing occurred after the administration of oral antibiotics. In both cases, only soft tissue inflammation was observed, with no involvement of the material used for cavity obliteration.

We observed three cases of recurrence and one residual cholesteatoma across the whole sample, accounting for 11% of the treated patient, with one case in BAG and two in sticky bone without the demineralised bone use group. Sorour et al. found no recurrence of cholesteatoma at the 12- and 36-month follow-ups among 20 patients after posterior wall sacrifice and obliteration [13]. In their study on 67 patients in which BAG-S53P4 was used for obliteration, De Veij Mestdagh et al. reported recidivism or the recurrence of cholesteatoma in only 6% of patients [36]. We attributed our results of cholesteatoma recurrence to the difficulty in precise obliteration of the epitympanum, in which the retraction pocket was created and then the cholesteatoma was formed. At the same time, the formation of a small tympanic cavity with a small volume of air capacity might make it susceptible to high pressure changes and the formation of retraction pockets. In order to prevent the formation of retraction pockets, we recommend that the tympanic membrane be supported by cartilage and precise epitympanum obliteration. Similar to Sorour et al., we observed no fistulas in the posterior wall of the EAC in our study group [13]. As previously reported, fistula formation was more common after canal wall reconstruction in CWD surgery when a titanium implant or a silicone block was used [11,39].

### 4.4. Cost/Duration of the Procedure

In terms of the costs of the obliterative materials, BAG-S53P4 was the most expensive: USD 450 (EUR 420) for 2.5 mL. The cost of using demineralised bone was USD 90 (EUR 80) per 1 mL. However, it should be emphasised that BAG-S53P4 is available in a ready-to-use formulation. Thus, compared with sticky bone, it is easier and shortens the surgery time. However, due to the limited durability of sticky bone from the moment of its preparation to its use in ear surgery, careful planning of the surgical steps is required when using sticky bone in ear surgery.

### 4.5. Limitations of the Study

Despite promising results, this study had some limitations. One of them was the small group of patients studied, but it should be noted that these are preliminary studies and antecedent multicentre studies. It should be noted, however, that the procedures were performed by one surgeon, which suggests the repeatability of the surgical technique in each case. The main objective of the study was to evaluate the effectiveness of the sticky bone technique as an innovative, and at the same time, cheaper method and to refer it to BAG as a reference obliterative material. The group of patients with BAG was the smallest, but the results obtained by the surgeon were comparable to those of other authors when assessing larger groups of patients, confirming appropriate surgical skills [13,14,15].

## 5. Conclusions

Sticky bone as well as BAG-S53P4 obliteration after the CWD procedure enables reconstruction of the conical shape of the EAC with a volume similar to that of a healthy ear. This is achieved due to the high plasticity of the materials used. Both techniques seem to have a minimal risk of complications and result in the achievement of a dry, self-cleaning EAC cavity. In the case of sticky bone, studies including larger numbers of patients are needed to determine the effectiveness of mastoid cavity obliteration and the occurrence of complications with respect to the proposed method.

## Figures and Tables

**Table 1:** Characteristics of the entire study group of patients according to the type of surgical procedure performed.

Characteristic		All	Material Used for Obliteration			*p*-Value
			Bioactive Glass	IPRF + Bone Dust Only	IPRF + Bone Dust + Demineralised Bone	
n		28	7 (25%)	12 (43%)	9 (32%)	
Gender	Women	12 (43%)	3 (43%)	7 (58%)	2 (22%)	0.342
	Men	16 (57%)	4 (57%)	5 (42%)	7 (78%)	
Age	Mean ± SD	45.6 ± 15.8	47.3 ± 22.0	41.7 ± 14.2	49.6 ± 12.6	0.517
Surgery side	L	17 (61%)	4 (57%)	8 (67%)	5 (56%)	0.892
	R	11 (39%)	3 (43%)	4 (33%)	4 (44%)	
Tympanoplasty type (Wullstein classification)	1	1 (4%)	0 (0%)	1 (8%)	0 (0%)	0.796
	2	9 (32%)	3 (43%)	3 (25%)	3 (33%)	
	3	15 (54%)	3 (43%)	6 (50%)	6 (67%)	
	4	3 (11%)	1 (14%)	2 (17%)	0 (0%)	
Bone dust volume (mL), n = 21	Mean ± SD	1.9 ± 0.8	-	2.1 ± 0.7	1.5 ± 0.7	-
Demineralised bone volume (mL), n = 9	Mean ± SD	2.2 ± 0.7	-	-	2.2 ± 0.7	-
All obliterated material volume (mL)	Mean ± SD	2.8 ± 0.9	2.7 ± 0.6	2.1 ± 0.7	3.8 ± 0.5	**<0.001**

**Table 2:** Results of surgical treatment depending on the type of procedure performed.

Characteristic		All	Material Used for Obliteration			*p*-Value *	*p*-Value #
			Bioactive Glass	IPRF + Bone Dust Only	IPRF + Bone Dust + Demineralised Bone		
n		28	7 (25%)	12 (43%)	9 (32%)		
Retroauricular area	Hole/depression	7 (25%)	4 (57%)	1 (8%)	2 (22%)	0.062	**0.043**
	Fully healed	21 (75%)	3 (43%)	11 (92%)	7 (78%)		
Retroauricular fistula	No	26 (93%)	6 (86%)	11 (92%)	9 (100%)	0.714	0.444
	Yes	2 (7%)	1 (14%)	1 (8%)	0 (0%)		
EAC	Hole, pocket	7 (25%)	1 (14%)	4 (33%)	2 (22%)	0.595	0.353
	Conical and smooth	20 (71%)	5 (71%)	8 (67%)	7 (78%)		
	Narrow and smooth	1 (4%)	1 (14%)	0 (0%)	0 (0%)		
TM	Healed with retraction pocket	2 (7%)	1 (14%)	1 (8%)	0 (0%)	0.879	0.594
	Perforation	1 (4%)	0 (0%)	1 (8%)	0 (0%)		
	Fully healed	25 (89%)	6 (86%)	10 (83%)	9 (100%)		
Cholesteatoma recurrent/residual	No	25 (89%)	6 (86%)	10 (83%)	9 (100%)	0.588	0.724
	Yes	3 (11%)	1 (14%)	2 (17%)	0 (0%)		
Merchant scale	0	28 (100%)	7 (100%)	12 (100%)	9 (100%)	-	-
EAC volume in operated ear	Mean ± SD	1.7 ± 0.5	1.4 ± 0.4	1.9 ± 0.6	1.7 ± 0.3	0.130	0.081
EAC volume in contralateral ear	Mean ± SD	1.1 ± 0.3	1.1 ± 0.2	1.2 ± 0.4	1.1 ± 0.2	0.584	0.829
Volume difference	Mean ± SD	0.6 ± 0.4	0.3 ± 0.5	0.6 ± 0.4	0.6 ± 0.2	0.171	0.058

* for comparison of the three groups; # for comparison of the bioactive glass and bone dust group (IPRF+ bone dust only, and IPRF + bone dust + demineralised bone).

## Data Availability

All data is available for the corresponding author request.
